# Resurrecting Gaia: harnessing the Free Energy Principle to preserve life as we know it

**DOI:** 10.3389/fpsyg.2023.1206963

**Published:** 2023-06-15

**Authors:** Caspar Montgomery, Inês Hipólito

**Affiliations:** ^1^Berlin School of Mind and Brain, Humboldt-Universität zu Berlin, Berlin, Germany; ^2^Department of Philosophy, Macquarie University, Sydney, NSW, Australia

**Keywords:** Free Energy Principle (FEP), Gaia Hypothesis (GH), active inference, biophilia virtue, ecological crisis, nature connectedness

## Abstract

This paper applies the Free Energy Principle (FEP) to propose that the lack of action in response to the global ecological crisis should be considered a maladaptive symptom of human activity that we refer to as *biophilia deficiency syndrome*. The paper is organised into four parts: the characterisation of the natural world under the Gaia Hypothesis, the employment of the FEP as a description of the behavior of self-organising systems, the application of the FEP to Gaia to understand coupling dynamics between living systems and purportedly non-living planetary processes, and the offering of positive interventions for addressing the current state of ecological crisis under this framework. For the latter, we emphasize the importance of perturbing stuck states for healthy development, and the necessary appreciation of life existing as nested systems at multiple levels in a hierarchy. We propose the development of human biophilia virtue in accordance with the FEP as a practical intervention for treating *biophilia deficiency syndrome* and helping to safeguard the balance of planetary processes and the integrity of living systems that depend on them, offering some examples of what this might look like in practice. Overall, this paper provides novel insights into how to catalyse meaningful ecological change, proposing a deliberate and disruptive approach to addressing the dysfunctional relationship between humans and the rest of the natural world.

## 1. Introduction

The relation of humanity to the rest of the natural world is essentially one of dependence. Our continued survival and flourishing are utterly contingent upon the state and functioning of our environment, which supplies us with eatable food, drinkable water, and breathable air. In the most practical sense possible, we are existentially bound to the rest of nature. Moreover, extensive empirical evidence demonstrates that connection to natural environments is a key ingredient of mental health. Specifically, spending time in natural settings has been shown to reduce stress, anxiety and depression, and enhance mood, cognitive function, mental health and wellbeing (White et al., 2019; Bratman et al., 2021). The psychological construct “connection to the world” correlates with therapeutic outcomes in treatment for depression (Watts et al., 2022), while Berto (2014) found that even exposure to images of natural scenes can improve mood and cognitive function. These results suggest that nature can be a powerful tool for promoting mental health and wellbeing.

However, to frame the value of nature purely in such utilitarian terms would be to gravely miss the point. Recent research in cognitive science suggests that humans possess an innate cognitive disposition towards nature, known as “biophilia.” This concept, coined by biologist Wilson (1984), purportedly has deep roots in our evolutionary history as the natural psychological disposition of humans to seek out and connect with other living organisms and natural environments (Clowney, 2013; Olivos-Jara et al., 2020; Barbiero and Berto, 2021). This evolutionary adaptation is believed to be the biological basis for human values of nature and environmental virtue (Clowney, 2013): the sense of connection and identity with, and fondness for (the continued existence of) all forms of life on Earth entails a sense of care towards the rest of the natural world and a willingness to protect it (Rowlandson, 2015). The concept of biophilia is therefore not merely a psychological theory, but rather a fundamental aspect of human nature that has played a crucial role in our survival and adaptation throughout our evolutionary history. The flip-side to this bond is the increasing prevalence of eco-anxiety among young adults and children upon learning about climate change, and observing human inaction towards it (Pihkala, 2020; Baudon and Jachens, 2021; Gunasiri et al., 2022). The question remains as to whether there can be an effective therapeutic strategy that addresses eco-anxiety without addressing the fundamental ecological crisis, or whether they are two crises which can only be tackled together.

We therefore arrive at a philosophical dilemma. On the one hand, human beings are imbued with biophilia, the “innate emotional affiliation … to other living organisms” (Wilson, 1993; p. 31). This biopsychological disposition suggests that people would be inclined to strive to protect and preserve the environment, including mitigating and ideally overcoming the threats of ecological breakdown. On the other hand, the lack of action to address the ecological crisis appears contradictory to this biophilic inclination. As our population and global footprint continue to expand, humanity's catastrophic impact on the Earth has become increasingly apparent: it is by now beyond doubt that activities such as deforestation, pollution of the oceans, and greenhouse gas emissions are harming our planet.

This paper seeks to address the contradiction between the biophilic inclination towards environmental protection and the lack of action taken to address the current ecological crisis. The paper proceeds in four parts. First, the natural world is characterised by the Gaia Hypothesis (GH). Second, the Free Energy Principle (FEP) is described, and then, third, employed to further develop and augment the GH. Fourth, it is argued that human failure to act on the ecological crisis does not invalidate the FEP. Rather, it is shown that this failure is indicative of an imbalance between active and sensory states, which reflects an unhealthy condition in the human species. If not addressed, this condition will lead to the dissipation of adaptation, the painful decline of our species, and ultimately the end of (human) “life as we know it” (Friston, 2013). The paper concludes by proposing that organised action motivated by biophilia in line with the FEP is necessary to treat this unhealthy condition.

## 2. The Gaia Hypothesis

“*We have to use the crude tool of metaphor to translate conscious ideas into unconscious understanding.”* (Lovelock, 2007, p. 178).

**Figure 1:**
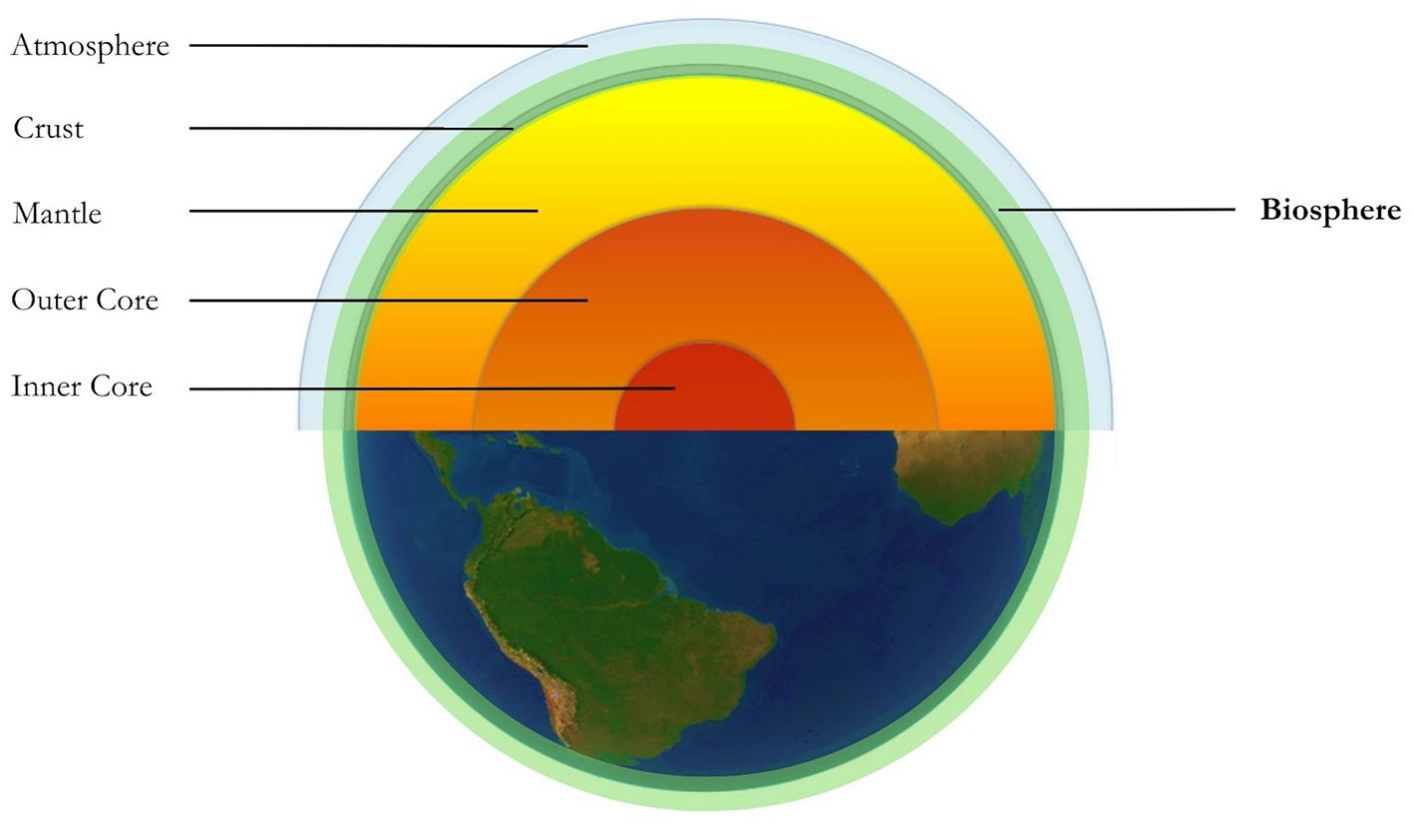
Selected layers of Planet Earth. The biosphere, highlighted here in green, encompasses the Earth's crust, the lands and oceans on its surface, and extends out towards the edge of the atmosphere.

The Gaia Hypothesis—named after the goddess who personified Earth in Ancient Greek mythology (Lovelock, 1972)—holds as its central tenet that the conditions required for life on our planet are maintained *by and for* the biosphere (Lovelock and Margulis, 1974). The “biosphere” (synonymous with “Gaia” hereafter) refers to the thin spherical layer of the planet at which life exists, starting where rock meets magma roughly 100 miles below the surface, and extending another 100 miles towards the thermosphere where air meets space (Lovelock, 2007; p. 19; see Figure 1). Consisting of rock, soil, water, air, and all of the ~5.5 x 10^14^ kg of organic matter in the known universe (Bar-On et al., 2018), Gaia is a *geophysiological* entity, since the totality of life and its non-living environment are bound as a single interdependent system (Lovelock, 1989). The “abiotic” parts are considered part of the total living organism, like the shell of a snail (Lovelock, 1972).

The GH is motivated by the fact that during the 3.8 billion years that life has persisted on Earth, conditions have remained remarkably constant and favourable across a huge range of parameters. Most obvious of these is temperature, which has remained within the tight parameters required for life to survive and thrive, despite an increase of the sun's output by at least 30% since life began (Lovelock and Margulis, 1974). The same applies to the pH and salinity of the ocean, despite the incessant depositing of salts from the land into the ocean by rivers (Lovelock, 2007; p. 34), and to the chemical composition of the soil and atmosphere (Lovelock and Watson, 1982). For a quarter of the time the universe has existed, these and countless other variables have been vastly different from what they “should” be, lying far from equilibrium at the levels that just happen to support life, even withstanding countless catastrophic volcanic eruptions, solar flares and asteroid strikes (Lovelock, 1979; p. 33). “For this to have happened by chance,” originator James Lovelock says, “is as unlikely as to survive unscathed a drive blindfolded through rush-hour traffic.” (Lovelock, 1989, p. 10).

The Gaia Hypothesis instead takes this constancy to be a natural product of evolution. However, in a modification of canonical Darwinism, rather than the evolution *of* individual organisms *to* an inert environment, “what evolves is the whole Earth system with its living and non-living parts existing as a tight coupled entity.” (Lovelock, 2007, p. 178). This can be understood through the “hologenome” concept (Rosenberg and Zilber-Rosenberg, 2011): according to this framework, a holobiont—a living host plus all of the smaller organisms living in dependence to it—is the unit of selection, such that changes in environmental parameters can lead to long-term adaptive changes to the holobiont via iterative changes to the constituent living systems. This is corroborated by Pricean formalisations, which demonstrate that natural selection can be extended to accommodate evolution (of Gaia) without reproduction (Bourrat, 2023). Thus, in a neo-Lamarckian turn within a Darwinian framework, the combined genetic wealth of information of the whole system (the hologenome) *acquires characteristics* that are conducive to its continued existence and in turn affect changes on the environment.

The biosphere thereby constitutes a complex system which “appears to have the … goal of regulating the climate and the chemistry at a comfortable state of life.” (p. 19). This fact was recognised by the scientific community in the 2001 Amsterdam Declaration, where it was acknowledged that the Earth is indeed a self-regulating system (Moore et al., 2001): just as an animal employs homeostatic feedback cycles to maintain viable conditions for its survival, the biosphere is kept within the limits needed for its own continued existence by thermodynamic imperatives (Karnani and Annila, 2009). This self-regulation tends not towards set points but adapts in a flexible manner to support the particular current life forms given the current environmental conditions (Lovelock, 2007, p. 19).^1^

Some literature on simulations can elucidate this further. “Daisyworld” was originally a caricature of Gaia wherein the only life forms—black and white daisies—absorb and reflect light, respectively (Watson and Lovelock, 1983). Temperature-driven competition between the two creates a balance in populations and therefore albedo effects, such that a planetary temperature favouring daisy growth is maintained, even as the output of the nearby “sun” varies in the simulation (Watson and Lovelock, 1983). Considered as a complex adaptive system, Daisyworld thereby exhibits “emergent self–regulation as a consequence of feedback coupling between life and its environment” (Lenton and van Oijen, 2002).

More recent models have corroborated and extended these findings. Using standard methodology from quantitative genetics, it has been shown that the self-regulating dynamics are explainable purely in terms of the low-level evolutionary dynamics of competition between the daisies: no higher level principle (such as teleology) need be invoked (Wood and Coe, 2007; Makarieva, 2022; Bourrat, 2023), at least at certain timescales (Weaver and Dyke, 2012). The implications have also been corroborated by models of different properties of organic systems: for instance, the metabolically abstract microorganism system (METAMIC) model simulates nutrient recycling (rather than temperature regulation) given appropriate thermodynamic constraints, and largely recapitulates the relevant behaviour of Daisyworld (Downing, 2002, 2003). Further, models based on based on Chemical Organization Theory (COT) and the Zero Deficiency Theorem (ZDT) demonstrate the autopoietic properties of the biosphere, and suggest how these might relate to other intrinsic features of living systems, such as autonomy and anticipation (Rubin et al., 2021). Finally, Daisyworld has been used in spatial systems dynamics simulations which demonstrate the importance of spatio-temporal interactions in such systems, thereby bridging the gap between purely abstract models and the three-dimensional coupling involving the biosphere (Neuwirth et al., 2015).

At a broader level, the perspective that the GH brings is appealing for many reasons. Conceptualised as a single complex system, Gaia changes in a way that is *adaptive* and *non-linear* (Lovelock, 1979); it displays *dynamical, emergent*, and *sudden tipping* behaviour: when bifurcation points are reached, new attractor states form, and higher order is established (Lovelock, 1979, 1989; Gleick, 1987; Capra, 1997; Lenton et al., 2020b);^2^ importantly for our purposes, it is *self-organising* (Lenton and van Oijen, 2002), and strong emphasis is given to the extreme *interdependence* between the processes of the system (Capra, 1997). Due to this combination of properties, the GH has both informed and been informed by a wide range of fields including ecology, climate science, cybernetics, complex systems theory, chaos mathematics, and the philosophy of mind.

Furthermore, there is an intuitive—and, indeed, poetic—attraction to Gaian thinking, as it allows us to transcend conventional levels of analysis, bridge philosophy and science, and prompt novel discussions on planetary-scale issues (Ruse, 2013). We generally find it intuitively easy to distinguish life from non-life, but only at the (somewhat arbitrary) level of resolution that the range of our senses allows: we would not recognise an *E. coli* bacterium without a microscope (Lovelock, 2007; p. 174). Similarly, although we struggle to see it that way (Lenton et al., 2020a), the natural way for aliens observing the Earth from space would plausibly be not to see a writhing mass of individual organisms on a dead planet, but rather a rock that has come to life (Oliver, 2020; p. 11). As a mode of thought, this visualisation of Gaia, while not *necessary* to comprehend it, provides a powerful tool for scientific understanding (de Regt, 2014). Thus, like the “overview effect” experienced by astronauts viewing our planet from orbit (White, 2014), the GH offers a glimpse of a fundamentally different way of thinking that in fact dominated most of human history, whereby the world was considered a single living organism—a “Thou” rather than an “It” (Frankfort et al., 1960). This may amount to a revival of some important elements of animism in its most basic form: a sensed appreciation of the vitality of the more-than-human aspects of Earth (Abram, 1997).

However, the GH has been subject to a wide range of criticisms (see e.g., Ruse, 2013; Rubin and Crucifix, 2022). Of the two most vociferous objections, however, the first worries unnecessarily that the use of language in the formulation of the idea entails commitments concerning the divine, sentient, or otherwise supernatural status of Gaia. Despite being largely compatible with strong claims such as those made in panpsychism, and somewhat ontologically slippery as an edge case of metaphysical categories, Lovelock (2007; p. 20) stresses that his illustrative goddess metaphor is just that.

Secondly, it has variously been argued that the GH is either false or unfalsifiable (Kirchner, 1989). Both cannot be correct, and, while it clearly contains and presupposes certain empirical claims (concerning the age of the solar system, the composition of the atmosphere, and so on), the actual idea of the biosphere as a self-regulating system is not a “hypothesis” at all but a *perspective*, a way of describing the fact of life's existence on Earth (though we will stick to its conventional name to avoid confusion). As per the assertions of Elgin (2019), truth is defined *within* this framework, rather than applied *to* it, and the mode of thinking that the GH engenders “refracts” with other modes, rather than competing with them. This is the “explanatory pluralism” that Kleinhans et al. (2010) refer to in their conclusion from an analysis of the philosophy of earth science.

Like Newtonian physics or psychoanalysis, the GH is therefore much more a framework or a method of investigation than it is a falsifiable empirical claim, and it is therefore to be judged by its *usefulness*, not its purported correctness, where usefulness is determined by factors such as parsimony, coherence and consistency, plus its power to explain phenomena, produce new facts, and so on, in a given context and a given time-frame (de Regt and Dieks, 2005). Thus, we effectively adopt a broadly pragmatic epistemological view of scientific understanding with regards to GH, similar to that endorsed by de Regt (2009) and others. By analogy, when meeting someone on the street, we conventionally treat the object of the encounter as a single fellow living being, rather than a composite menagerie of over 40 trillion discrete microorganisms in a state of cohabitation (Sender et al., 2016). This “Human Hypothesis” is not more or less correct than a “Microorganism Hypothesis,” it is simply a different (and usually more useful) scale at which to engage with the phenomena. In principle, then, the GH can at worst be called unpragmatic, but not false.

## 3. The Free Energy Principle for self-organising interacting systems

In this section, we aim to present the fundamental framework of the FEP and active inference. Subsequently, we intend to apply this framework to the planetary-scale processes which comprise Gaia. By doing so, we can apply the FEP to analyse the interdependent dynamics between living systems and (non-living) planetary processes. Specifically, this framework allows us to gain insights into future states of Gaia, formulate predictions, and devise potential interventions to alleviate future dysfunction.

The second law of thermodynamics, which governs the behaviour of energy and entropy in a system, stipulates that all open systems—which means almost all systems in the natural world—tend to dissipate, i.e., tend towards chaos or increased entropy. For example, a cup of hot coffee will always cool down to room temperature, but it will never spontaneously heat back up to its original temperature. The principles of thermodynamics in physics stipulate that the behaviour of open systems is critically influenced by the interaction with the environment. The flow of these interactions cannot be replicated in the opposite direction due to the irreversible nature of time (Von Bertalanffy, 1950; Ptaszyński and Esposito, 2019; Pokrovskii, 2020; Rovelli, 2023).

However, there are displays of “negentropy” (Schrödinger, 1944), pockets of the universe where order and bodily integrity are maintained in the face of the surge towards chaos. These pockets are self-organising systems, or “*things*” (Hipólito, 2019), including—but not limited to—living organisms. These *things* resist entropy by interacting with the world such that their integrity is maintained, i.e., the process of homeostasis. They use energy to keep themselves within the restricted set of possible states that allows for their continued existence. Hence the workings of feedback cycles—drinking when one is thirsty, retreating from a hot fire, etc.—represent *work* that can only be done by the energy that is “bound” within the system and therefore useful, whilst the remainder is called “free” energy (Friston and Stephan, 2007). Simply by existing, then—by virtue of seemingly defying the second law of thermodynamics by acting to minimise entropy—all such self-organising systems necessarily act so that their free energy is minimised. This is the Free Energy Principle.

Moreover, a system's free energy bears upon various other related concepts in addition to entropy, including the number of possible states the system can manifest, the predictability of its behaviour, and the level of “surprise” associated with new data (Table 1).^3^ Furthermore, at least in Karl Friston's formulation,^4^ free energy is equal to *information*, i.e., the number of new binary facts about the state of the world gleaned by a system from a given data sample (Solms, 2021). This means that, adhering to the FEP, every *thing* should act so as to minimise the amount of information about the world it needs to process, which amounts to minimizing its free energy, chaos or entropy and thus maintaining its physical integrity.

**Table 1:** The relationship between Friston's free energy and related quantities.

**Free energy**	**Low**	**High**
Bound (i.e., useful) energy	High	Low
Entropy	Low	High
Number of possible states of the system	Low	High
Predictability	High	Low
Surprise	Low	High
Average information (i.e., uncertainty) of a series of measurements	Low	High
Mutual information between internal and external states	High	Low

Organisms minimise free energy by action. Active inference is a corollary of the FEP that allows us to model and understand a complex system's behaviour. In its formulation, living systems:

will *appear to engage in active Bayesian inference*. In other words, they will appear to model—and act on—their world to preserve their functional and structural integrity, leading to homoeostasis and a simple form of autopoiesis (Friston, 2013; p. 1, emphasis added).

“Active inference” is a modelling technique that, because it employs a scale-free formalism known as Markov blankets, allows us to understand and make predictions about the coupling dynamics taking place between interacting systems. A living system is equated with “internal states,” while the system it interacts with is “external states.” Because, mathematically, internal and external states are conditionally independent, they do not directly influence one another. The direct influence occurs via yet another set of states: active and sensory states. A balanced reciprocal influence between active and sensory states is fundamental for a system (internal states) to maintain a healthy interaction with—and thereby adaptation to—its environment (external states).

These interdependencies and dynamics are understood by employing a Markov blanket. A Markov blanket is a statistical tool that can be applied to any system that self-organises. More precisely it furnishes probabilistically defined tools to set a system's boundaries through conditional dependence or independence relationships. Because they are scale-free, they can be applied to any level of analysis of the natural world. The concept of Markov blanket involves defining a set of variables, denoted as b, that surround the internal states while labelling all other external variables as η.

The internal and active states are a function of a system's internal and blanket states. Similarly, external and sensory states are a function of external and blanket states. This sparse dynamical coupling means that the state of a system at a moment in time results from the interactive dynamics between internal, sensory and active states; meanwhile, the state of the environment at a moment in time is a result of the dynamics between external, sensory and active states. It follows from this that internal and external states reciprocally (indirectly) influence each other.

**Figure 2:**
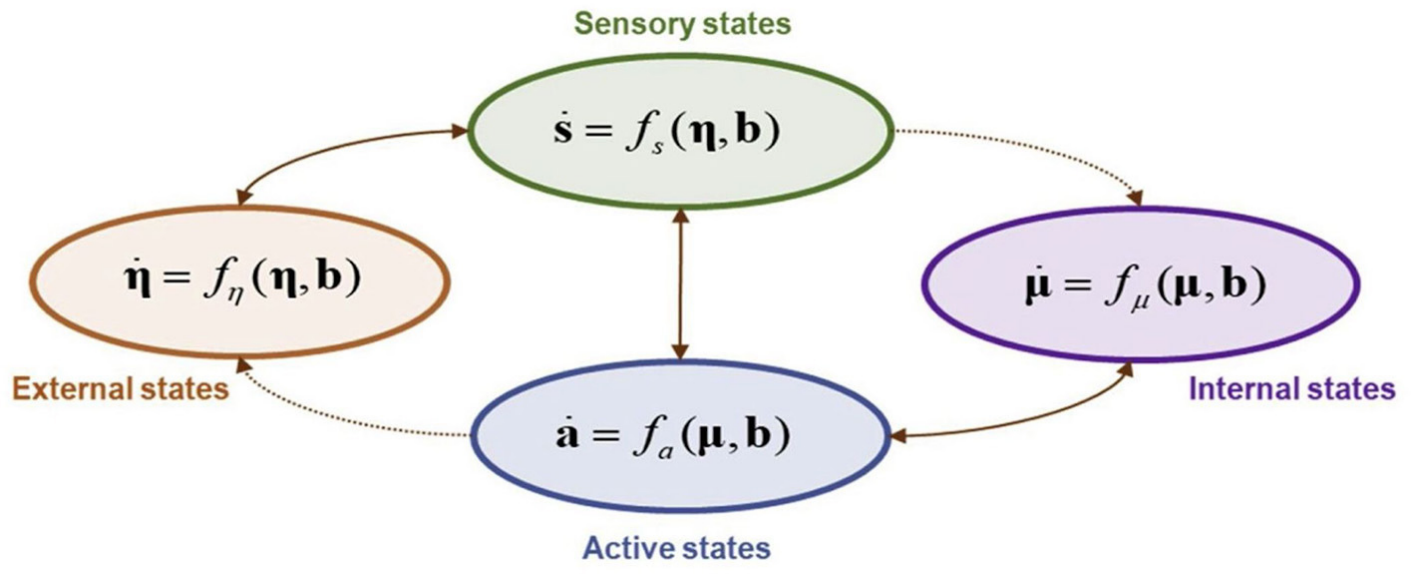
The partitioning of a system into states. The result is composed of internal (purple) and external or hidden (orange) states, separated by a Markov blanket consisting of sensory (green) and active (blue) states. The schematic highlights directed influences with dotted connectors. Autonomous states are those not influenced by external states, while sensory and autonomous states constitute an entity, namely, blanket and internal states. A system of scientific interest (e.g., a cell, an organ, an organism, a community, an ecosystem) is composed of sensory, active, and internal states, as described in more detail by Hipólito (2019).

There are therefore two ways for a *thing* to minimise its free energy, and thereby maintain its own integrity and continued existence: the first is to *change the model* (or belief) that it instantiates so as to more closely resemble the world, by perpetually generating, testing and updating it based on incoming prediction error information. The second is to *change the world* by acting upon it to bring it in line with predictions, under the generative model. These processes form a feedback loop, from within the system to without and vice versa (as seen in Figure 2).

**Figure 3:**
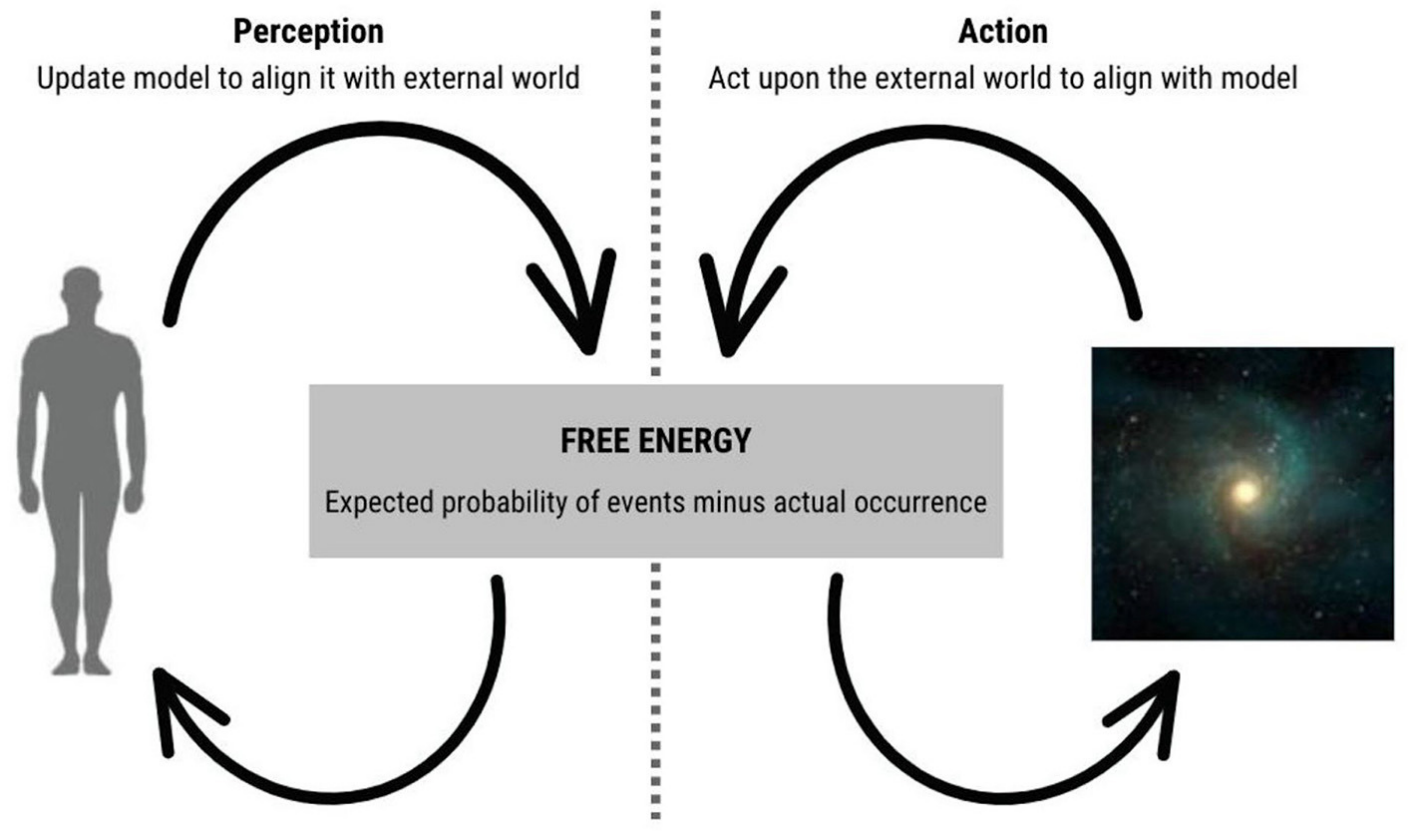
An organism-environment interaction. Active inference and the Free Energy Principle describe a closed causal loop of action and perception within a system, such as a human. This loop involves the system using inference to both update its model of the environment and act upon it.

This loop involves the system being described as if using inference to both update its model of the environment and act upon it, as illustrated by Figure 3 in the case of a human. The loop can be described in terms of the following steps (where we can regard free energy as the total amount of prediction error):

1. The generation of a prediction of its sensory input based on its internal model. This prediction is compared to the actual sensory input to generate a prediction error.
2. The updating of its internal model to reduce the prediction error. This involves adjusting the probabilities assigned to various possible causes of the sensory input.
3. Based on an updated internal model, the system is then supposed to generate an action that it “believes” will lead to a predicted outcome in the environment.
4. The system's action upon the environment is seen as generating new sensory input.
5. The loop then repeats, as a system generating a new prediction of sensory input based on the updated internal model, and so on.

The system is then understood by scientifically interpreting and predicting its behaviour as a system's internal model is constantly updated in response to new sensory input to improve the accuracy of predictions. This loop therefore minimises the system's uncertainty about the causes of its sensory input, since it is driven by (or amounts to) the minimisation of free energy.

## 4. Applying the Free Energy Principle to the Gaia Hypothesis

In this section, we utilise the FEP to elucidate the coupling dynamics between biological systems and geophysical planetary processes, which make up the biosphere. In this picture, organisms and ecosystems are self-organising systems which are each part of a larger multiscaled system. The FEP framework facilitates greater understanding of current states and enables us to make predictions about future states. Additionally, it helps to identify and develop interventions to rectify any maladaptive responses, namely actions which are antithetical to maintaining the integrity of a system: human behaviour that leads to an imbalance within the Markov blanket states is therefore to be considered maladaptive or pathological.

As a self-organising system, Gaia meets the requirements to be formalised as part of an active inference system. It exists in *non-equilibrium steady states* (NESS), viz. its processes and states must persist over time within a range of states that are far from equilibrium (i.e., resisting entropy); and it is *distinguishable* from its larger scale cosmic environment, defined by conditional independence between its internal and external states (which influence each other only vicariously through blanket states).

The issue of distinguishability—that is, the ability to differentiate the biosphere from the earth's core on one side and the larger-scale solar system, galaxy, and universe on the other—presents a challenge. Experts from diverse fields, from climate and complexity scientists to Buddhist philosophers, have stressed that the biosphere is an integral part of the larger cosmic environment, demonstrating fundamental continuity with and interdependent upon it (McMichael, 1993; Egri et al., 1999; Danvers, 2016; Kaçar et al., 2021; Boulton, 2022; Stahel, 2022). However, while the ontological continuity of the Earth and the rest of the universe should be emphasised, it is nevertheless possible to employ epistemic (statistical) tools to help us understand the interactions between multiscaled systems, in the same way that we can usefully distinguish between cells and organisms or countries and continents without supposing that they are truly separable.

We can therefore use Markov blankets to elucidate the interdependencies and dynamics between the nested systems, since, as outlined above, a Markov blanket is scale-free, making it suitable for application across various scales in the natural world, depending on the object of scientific interest. For example, we can employ Markov blankets in a multi-scale system to further understand and predict the interactions between human behaviour, Gaia, and the Cosmos.

To envision what this might look like, we can draw from Rubin et al. (2020) who assigned Markov blankets specifically to the Earth's climate system. The authors begin by defining metabolic rates of the biosphere as internal states and the changes in “space weather” (mostly driven by solar radiation) as external states. Further, they define active states as the changes in greenhouse effects and the reflection of sunlight, and sensory states as ocean-driven changes in global temperatures. Internal and external states are conditionally independent, thereby indirectly influencing each other via the ocean's very slow reaction to thermal fluctuation. Through this formalism, “the Earth's climate system [can] be interpreted as an anticipatory system that minimises variational free energy” (Rubin et al., 2020).

**Figure 4:**
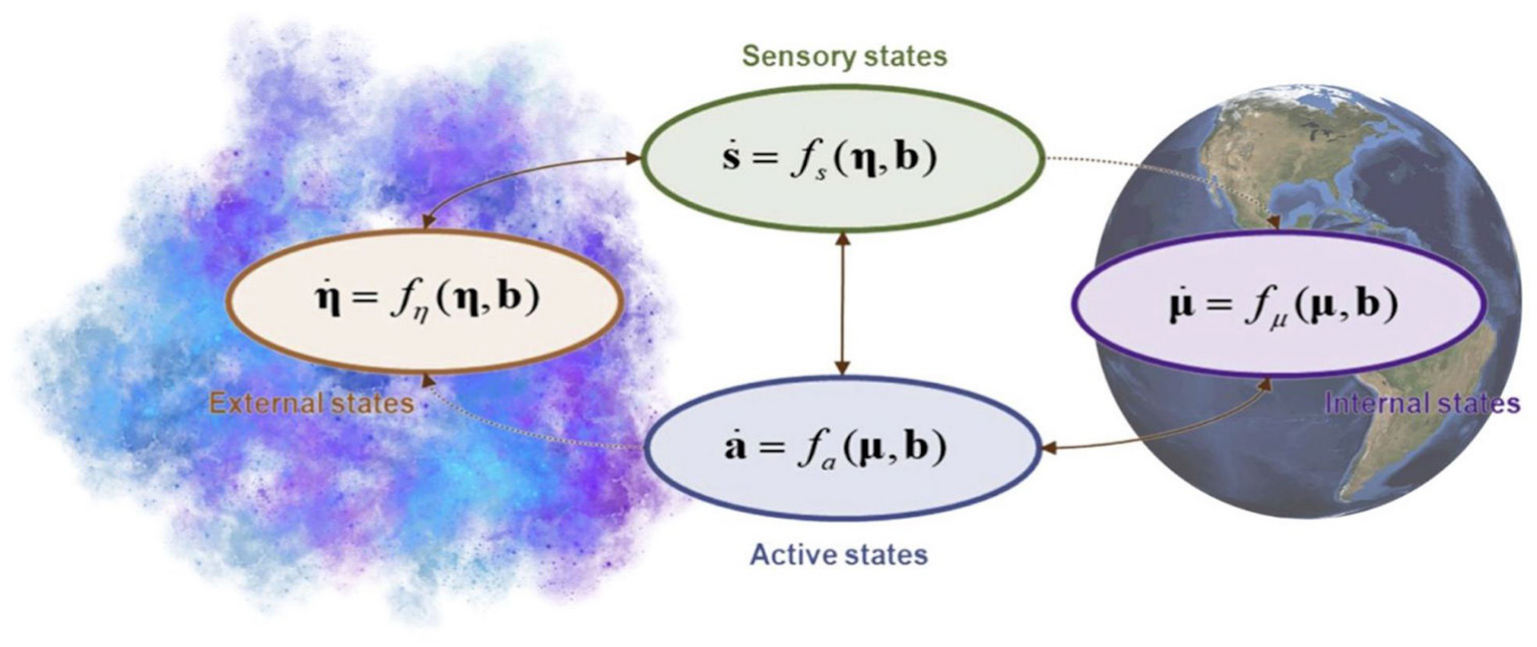
The Markov blankets of Gaia. When we consider the geophysical processes of the biosphere as *internal states*, the *external states* refer to everything outside the organism, including the atmosphere, other planets, and celestial bodies. *Sensory states* include gravitational effects of other celestial bodies, climate and weather, the evolution of life, the composition of the atmosphere. *Active states* are the Earth's gravitational effects, magnetic field, radiation etc.

As stressed previously, because Markov blankets are a scale-free formalism, we can broaden our object of scientific interest to cover the entire biosphere. If the geophysiological processes of the biosphere are considered internal states, then external states would refer to the external environment, which includes everything outside of the system, such as the outer atmosphere, and other planets and celestial bodies, as well as the molten core of the planet lying below the Earth's crust. Sensory states are the influences the external environmental states have on the planet Earth such as the impact on the Earth's rotation, orbit, and ocean tides by gravitational effects of the sun, moon, and other planets. Earthquakes and volcanoes caused by the influence of by convection currents within the mantle on plate tectonics; climate and weather are influenced by solar radiation and space weather events; cosmic events like meteor impacts can trigger evolutionary changes and cause mass extinctions; the composition of the Earth's atmosphere is similarly influenced by cosmic events. Active states are simply the capacity of the earth to assimilate external influence and adjust to it (i.e., change its properties) in ways that will feed back to the external states. For example, the Earth's gravitational force causes slight perturbations in the orbits of other objects in the solar system, and its magnetic field interacts with the solar wind, affecting phenomena such as the aurora borealis. The Earth also emits radiation that can be detected by other objects in the solar system, even from billions of kilometers away (Figure 4).

The active states of a system embody its primary mechanism for self-regulation, representing its efforts to remain within the bounds required for ongoing existence, based on predictions about the present and future states of the system. For instance, the current levels of greenhouse and albedo effects, soil pH, and the number of living organisms reflect the predictive attempts of the Gaian system to adapt to the anticipated effects of solar events, volcanic eruptions, ocean salinity changes, and other environmental factors in the future. It is in this context that the role of life itself assumes paramount importance in the self-regulation of the biosphere.

In information theory, the dynamics described above can be explained by considering the active states as realizing the prior predictions of the generative model, while the sensory states provide information resulting from the external world, which is used to update the inferred state of the biosphere into posterior beliefs. The system responds to prediction errors, to accommodate this new information, while at the same time acting on the external states to resolve the prediction error, to the extent possible. Note that only certain kinds of prediction errors can be actively resolved. In neurobiology, the prediction errors resolved by movement are the predictions of signals from the muscles, known as proprioception. If prediction errors cannot be resolved via action, then they will be resolved by changing predictions. This can be cast as Bayesian belief updating and an elemental kind of perception. Updating the priors to accommodate this new information causes a change in the macro properties responsible for enabling such fluctuations, thereby updating the model itself such that the mutual information between the internal and external states is maximised, and free energy is minimised. Describing Gaia as a complex system of interacting, self-organising processes that exist in non-equilibrium steady states, actively adapting to changing environmental conditions, affords new syntheses between different conceptual approaches. For instance, combining the greenhouse effect and the role of life in the biosphere provides a unique opportunity to incorporate energy and information as two aspects of the same underlying physical process (Hermann-Pilath, 2011), namely the minimisation of free energy.

Finally, a purported weakness of the FEP actually speaks in favour of its application to the GH.^5^ Raja et al. (2021) rightly point out that Markov blankets do not automatically capture every relevant property of biological systems: relational properties such as affordances—what the environment “offers the animal [sic], what it provides or furnishes, either for good or ill” (Gibson, 1979; p. 127)—fall into this bracket. However, the claim that they are precluded by the FEP is not true, at least with respect to the biosphere: on the contrary, affordances correspond well to Gaia as one rung in a ladder of *nested* Markov blankets—with the system in question at every level ranging up from, say, cells and organs, through animals and ecosystems, to our solar system (Dennison, 2020) and the whole cosmos. In this framework, it is entirely coherent to say that our solar system is one that *affords* a living planet, or that the geosphere *affords* a biosphere, when we consider that lower levels or layers in such hierarchies operate at much faster rates of change than higher levels (Wu, 2013). The affordances available in an environment are not determined solely by the physical properties of the environment, but also by the organism's goals, abilities, and previous experiences. In the case of the sun and its affordances for life on the planet, we can apply this theory to understand how the physical properties of the sun, as well as the goals and abilities of organisms on Earth, shape the affordances that the sun provides.

The same principles of dynamics, interdependence and scale apply to the symbiotic evolution and exchange of genetic information throughout the history of life (Paracer and Ahmadjian, 2000; Watson, 2002; Gontier, 2016), and, increasingly, to the tight coupling between humans and technology and the possible emergence of autonomous symbiotic AI systems (Wang et al., 2021). Hierarchy theory is therefore not only compatible with the FEP as applied to the Gaia Hypothesis, it is a highly tractable vehicle for understanding it, in terms of the complexity in ecosystems (Allen and Starr, 2017), the evolution of the planetary genome across billions of years (Margulis and Sagan, 1995) and changes to the (“non-living”) aspects of ecological landscapes (King, 1997) at different scales of time and space. Overall, there is good evidence that phenomena in earth science are emergent, but should be considered irreducible to the laws of physics (Kleinhans et al., 2010): we therefore aim to marry “bottom-up” principles intrinsic to the FEP from fundamental physics to the higher-level phenomena captured by the GH. As emphasised above, we are not arguing not that this approach to understanding life on Earth is the best one, merely that it can be beneficial.

## 5. Human behaviour as an aspect of Gaia

We can now employ Markov blankets to further understand and predict the actions of humans within the biosphere. Specifically, we want to understand the extent to which human behaviour is antithetical to the healthy behaviour of the larger system, why this is the case according to the FEP, and what might be done to improve the situation.

Larger systems provide the smaller nested systems they contain with existential challenges as well as affordances: the sun provides energy for photosynthesis and regulation of the climate, but also threatens humans with bad harvests and skin cancer. These challenges demand adaptation in a dynamic fashion. The mechanism for this—as per the FEP—is to assimilate information (in the form of prediction error) of an upcoming threat from the world, and act accordingly on the world such that the system's integrity will be maintained and free energy minimised. Thus, according to the FEP, living beings have a natural tendency to act upon their environment to prevent the dissipation of their integrity. Therefore, if the principle holds true, the knowledge that (for example) climate change caused by continued greenhouse gas emissions will have catastrophic consequences for continued human existence should prompt drastic action from the human species to rectify it, in the form of behaviour that reflects biophilia. The lack of such action can be seen as an imbalance within (Markov) blanket states, therefore indicating a profoundly unhealthy condition in the human species, whereby it is unable to maintain its integrity by adapting to changing environmental conditions. It is the combination of *knowing* that disrupting the balance in such a way is self-harming, and yet acting as if this were not the case that constitutes a pathological state.

According to the FEP, a system's interaction with the environment consists of using inference to both update the model of the environment and act upon it to make the environment fit the model.^6^ Therefore, learning (as we have done) that the global *status quo* represents a serious threat to human civilisation, one would expect to see behaviour change accordingly. This would amount to gathering more evidence and then acting upon it to minimise free energy and, by extension, preventing the rise of entropy, chaos, and the ultimate dissipation of life.

**Figure 5:**
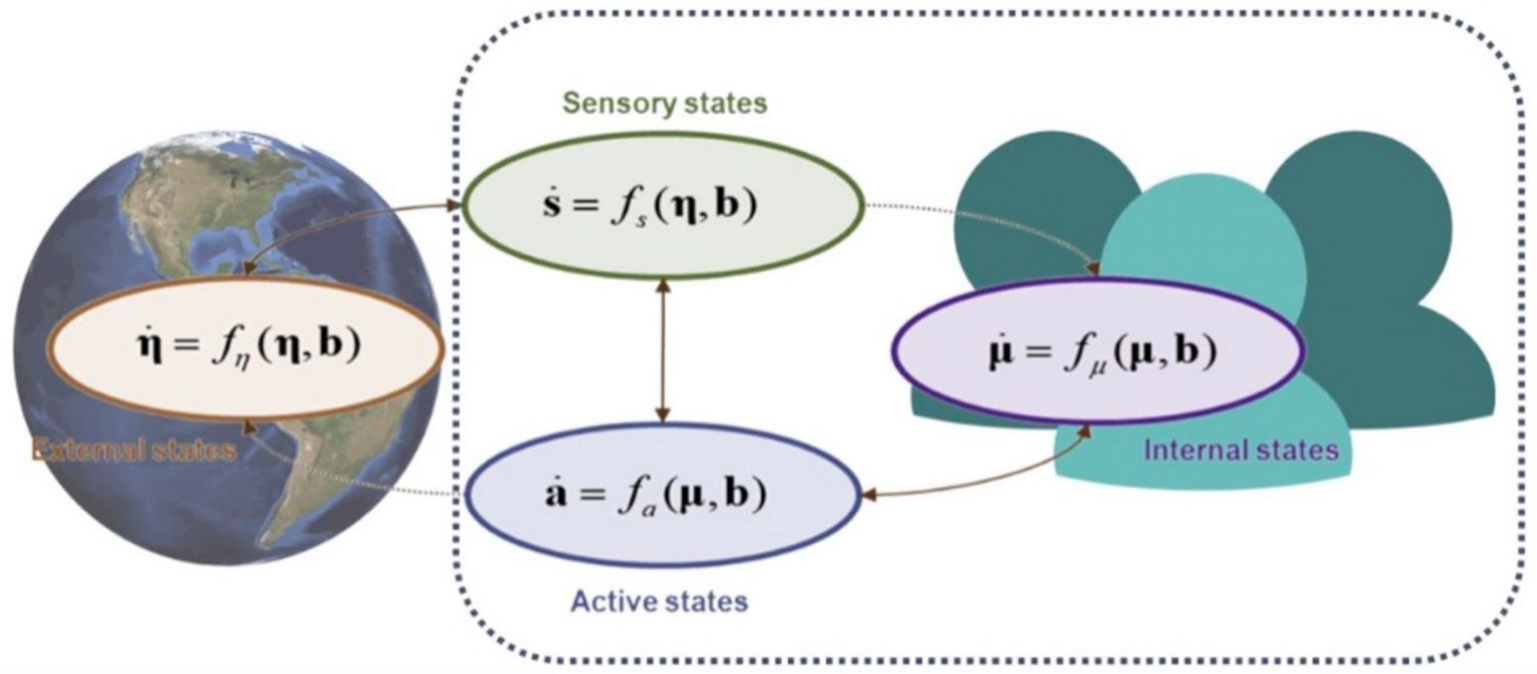
Imbalance in the Markov blanket. The figure shows influences occurring within the Markov blanket, which is marked by a dotted box and includes internal, active, and sensory states. This imbalance results in an insulation or echo-chamber effect in internal states that are impermeable to information from external states that demand action.

Instead, the human species either rejects the information available so as to remove the imperative for action or, possessing the information available, still refrains from taking action, or worse, continues the same harmful behaviour and perpetuates the same systems (such as capitalism) that enable it. If the FEP holds, then the observed contradictory behaviour is a maladaptive state, arising as an imbalance within the Markov blanket states (Figure 5).

Using the lens of the FEP to model the dynamics of the relationship between humans and the environment, it is possible to see the current lack of sustainability and pro-environmental behaviour as a pathology of the human species that must receive treatment. We label this condition *biophilia deficiency syndrome*. To unpack the notion of biophilia deficiency syndrome—and lend it construct validity—it is useful to consider other applications of the FEP to pathology. In general, these applications rest upon instances of false inference, of the sort found in psychiatry and neurology. For example, inferring things are not there when they are, describes certain dissociative and hysterical (e.g., neglect) syndromes. Conversely, inferring things are there when they are not, describes phenomena like hallucinations and delusions.

These pathologies are commonplace in computational psychiatry and generally reduce to a failure to assign the right weight or precision to prediction errors. Perhaps the most prescient example of this is Parkinson's disease, characterised by a failure to initiate movement or action that manifests as bradykinesia. From the perspective of the FEP, this is simply explained by a failure to attenuate evidence from sensors providing evidence that the one is not moving. Put simply, a failure to realise prior predictions can be due to a failure to ignore evidence that predictions are not coming true. This seems to be an apt description of our communal and cultural response to the ecological crisis. In other words, biophilia deficiency syndrome can be seen as a collective or cultural Parkinsonism that inherits from our inability to attenuate the evidence that we are not acting in a remedial or restorative fashion.

From the perspective of the FEP, this is a pernicious pathology because doing nothing is a Bayes optimal response in the face of a pathological attention to various sources of evidence. In computational psychiatry, one then is led to therapeutic interventions that rest either on restoring neurochemical deficits in the brain or engaging in therapy that allows people to become skilled in deploying their attention—and exploring other models of active engagement with the world. In short, enabling patients to escape from particular patterns of active inference in which they are stuck.

By leveraging the concepts of the FEP and active inference, a compelling analogous perspective on how humans relate to the ecological crisis emerges. The FEP and active inference formalisms shed light on climate change and the wider ecological crisis as a predicament that is both human-generated and often disregarded due to a lack of action. According to the FEP, living systems possess a biologically encoded inclination to interact with their environment in order to survive and adapt.

However, the current state of affairs diverges from this natural inclination. Despite climate change being a pressing issue, proactive measures to reverse its effects are not given the priority they deserve. This discrepancy between the expected and observed actions can be understood in the framework of the FEP as an illness. It implies that systems, including human societies, may form erroneous inferences that prevent them from effectively addressing the problem at hand.

Given this characterisation, it is imperative that we recognise the current ecological imbalance as a “stuck state” that requires active disturbance to bring about systemic change (for detail see Hipolito, 2023). What we are calling for amounts to a kind of “homeostatic awakening” (Wong and Bartlett, 2022): a deliberately-induced, disruptive shift in trajectory reflecting a prioritisation of planetary homeostasis over infinite growth. To achieve this, we must focus on addressing the reciprocal unbalanced influences between sensory and active states at multiple levels of nested systems, rather than solely at the level of the individual: the best efforts of individuals to change their own behaviour will be in vain so long as the lack of appreciation of dynamics at higher nested levels persists. On the other hand, if we can come to truly understand our relationship to Gaia at a depth that becomes part of cultural common sense,^7^ then we will no longer be able to deny our current knowledge, and more appropriate behaviour will follow as a natural reflection of our (FEP-driven) values. Meaningfully addressing the ecological crisis is therefore synonymous with restoring the felt connection between humans and the rest of life on Earth, such that we collectively come to truly embody the fact that we are a part of, not apart from, the natural world (Seth, 2022). This will entail the transformation of the very concepts of “self” and “nature” to reflect a part-to-whole relation, rather than a subject-object dichotomy, in much the same way that integrating the understanding that *the Earth revolves around the sun* required a shift in what those words actually mean (Kuhn, 1962).^8^

It should be clear that individual responses will not suffice for problems that are essentially collective and relational. Thus, beyond enforcing institutional climate commitments at the national and international levels, future work should focus on identifying strategies for action and intervention at community, societal and whole-species levels to stimulate both the integration of ecological knowledge and the practice of biophilia virtue. In this way, both sides of the Markov blanket are addressed simultaneously in an effort to remedy the insulation of internal states (i.e., to address the loss of connection between humans and the Earth; Figure 5). Providing a full manifesto for doing so is far beyond the scope of this paper; however, some intuitive examples of measures that can point towards directions of implementation and be taken at the institutional level to catalyse biophilia virtue as conceptualised under the FEP may include the following:

(1) *Education:* Ecopedagogy is a holistic approach to education that emphasises the interdependence of social, economic, and ecological systems. One of the main goals of ecopedagogy is to integrate environmental sustainability and social justice into educational practices and to encourage learners to take an active role in addressing environmental and social issues. By promoting experiential and place-based learning, ecopedagogy encourages learners to connect with their local environment and community, fostering a deeper understanding of ecological systems and their interrelationships (as opposed to the abstract intellectual knowledge many of us currently harbour). For example, learning about the fundamental role of fungi in the web of life through mycology can foster an appreciation of Gaia's interconnected nature and prompt a shift away from the anthropocentric insistence on thinking of organisms as individuals (Sheldrake, 2020), as well as providing practical skills in food-growing and restorative practices (Stamets, 2005).

Ecopedagogy also emphasises critical thinking skills and encourages learners to engage in social and environmental activism, by promoting, firstly, a true *reciprocity* between nature and humans (Varanasi, 2020) that recognises that the fate of the latter is dependent on the state of the former, and secondly, non-linear thinking, so that the Earth and its inhabitants can be encountered as the highly complex adaptive systems they are (Varela et al., 1991; Margulis and Sagan, 1995; Capra, 1997; Duncan, 2018; Fried and Robinaugh, 2020; Hayes and Andrews, 2020). Thus individuals and collectives can become more aware of the impact of human activities on the natural world at multiple levels of analysis, and develop a corresponding sense of responsibility towards the environment (Zysltra et al., 2014; Norat et al., 2016; Misiaszek, 2020; Hung, 2021). A shift in the value pyramid of the educational priorities towards sustainable behaviour not only acts as a perturbation to the *biophilia deficiency syndrome* but also addresses the raising eco-anxiety symptoms reported by young generations (Pihkala, 2020; Baudon and Jachens, 2021; Gunasiri et al., 2022).

(2) *Urban design*: Green spaces, such as parks, green roofs, or community gardens, provide opportunities for individuals to connect with nature, even in urban areas. These spaces offer a range of benefits, from relaxation and recreation to habitat for wildlife. Community gardens, in particular, can promote community engagement and healthy eating, while also fostering a deeper connection to nature through shared stewardship (Roe and McCay, 2021).

(3) *Technological and Artificial Intelligence design*: Technological design can be used to promote sustainability and reconnect humans with nature. This can be achieved through environmental monitoring to understand the impact of human activities on ecosystems (Bodini, 2012), nature-based gaming to promote education about biodiversity and conservation (Schneider and Schaal, 2018), and sustainable design of buildings and green infrastructure to create and maintain natural environments in urban areas (Restall and Conrad, 2015; Leavell et al., 2019; McKewan et al., 2020).

(4) *Mindful nature practices:* Mindfulness practices like yoga or meditation can help individuals develop a greater sense of embodiment and connection with their physical selves, which can translate into a felt appreciation of the “interwoven nature” of the natural world (Danvers, 2016). By cultivating mindfulness practices, individuals can become more attuned to the natural world and develop a greater sense of respect and responsibility towards the environment (Amel et al., 2009; Barbaro and Pickett, 2016). Conscious and responsible use of psychedelic plant medicines like ayahuasca and psilocybin can also be fruitful options. Psychedelic use predicts nature connectedness (Nour et al., 2017; Kettner et al., 2019), and solidarity with other species (Pöllänen et al., 2022), translating into pro-environmental behaviour (Forstmann and Sagioglou, 2017), as well as both concern for and objective knowledge about climate change (Sagioglou and Forstmann, 2022), while a recent survey on “psychedelically induced biophilia” found a tendency to elicit a “passionate and protective” connection with nature (Irvine et al., 2023). Engagement with these practices would also encourage interaction with cultures from which they originate, affording opportunities for learning from and cooperation with indigenous peoples who currently steward lands with 80% of the world's biodiversity (Watene and Yap, 2015), and increasingly carry the flame for cultural diversity and lost ecological wisdom (Toledo, 2013; Rowlandson, 2015; Etchart, 2017; George et al., 2019).

This list is neither fully developed nor exhaustive, but offered as an exemplar of how the principles provided in this paper can be applied. Despite efforts made by individuals, organisations, and governments to reduce carbon emissions and mitigate the impact of climate change and the wider ecological crisis, progress has been hindered by the multifaceted and global nature of this problem. To catalyse meaningful change, a deliberate, disruptive and holistic approach is necessary to address the symptomatic ways in which humans induce (self-)harm upon Gaia.

## 6. Conclusion

This paper employs the Free Energy Principle to argue that the lack of action taken in response to threats to planetary life, such as those posed by the ongoing ecological crisis, should be treated as a maladaptive disruption to Gaia's Markov blankets, referred to as *biophilia deficiency syndrome*. Adopting a pragmatic, pluralist epistemological approach to understanding planetary life processes, the paper proceeded in four parts. Firstly, it characterised life under the Gaia Hypothesis, wherein the biosphere constitutes a single self-organising system of living and non-living planetary processes, arguing that this is a plausible and useful way of looking at life on Earth. It then described the Free Energy Principle as a means of understanding self-organising (living) systems, before employing the FEP to elucidate the coupling dynamics within the biosphere with a view to developing beneficial interventions. Finally, the paper demonstrated the possibility for positive alternatives afforded by this framework to remedy the ongoing crisis of ecological breakdown, emphasising the importance of perturbing stuck states at multiple levels of nested hierarchies to promote biophilia virtue and, by extension, the healthy development of human behaviour as part of Gaia. Ultimately, this paper considers the Gaia Hypothesis through the lens of the Free Energy Principle to reveal insights into how we can restore the balance of planetary processes and safeguard the integrity of living systems that depend on them.

## Author contributions

CM conceived the initial idea for the paper and produced the first manuscript. IH revised the thesis and wrote additional sections of the manuscript. All authors generated figures, contributed to manuscript revision, and read and approved the submitted version.

## Footnotes

^1^For instance, oxygen, which was extremely poisonous to the organisms of the time, was virtually absent from the atmosphere for more than a billion years of life, until the explosion of aerobic life coincided with a dramatic increase of oxygen levels as it was produced by cyanobacteria (Schirrmeister et al., 2013; Lyons et al., 2014).

^2^These tipping points are partly what make climate change such a difficult challenge, especially since we won't necessarily know when they have been passed (Lenton et al., 2020b).

^3^Though a technical term, this formal meaning of ‘surprise' is close enough to its use in common parlance.

^4^As opposed to Helmholtz's and Gibbs' free energy, which refer to related thermodynamic and chemical quantities, respectively.

^5^Note that applications of the FEP to systems such as animals introduces the notion of planning (as inference) in a psychological sense and the consideration of epistemic and pragmatic affordances. However, here, we are applying the FEP to the systems that do not necessarily plan, such as the weather, market economy, evolution, et cetera.

^6^Or, fails to attenuate sensory evidence that nothing is — or can — be done about the situation.

^7^It is arguably only in WEIRD (Western, educated, industrialised, rich, democratic) cultures that this is not already the case: “Many indigenous belief systems share a view of people and nature as part of an extended ecological family … These different groups … separated by geography, culture and time, have described this phenomenon. The Māori worldview (te ao Māori) acknowledges the interconnectedness of all living and non-living things. The Raramuri worldview includes ‘Iwigara': the total interconnectedness of all life, physical and spiritual. From the point of view of these belief systems, *feeling separate from nature would signify a state of disconnectedness and constitute a significant rupture in wellbeing*.” (Watts et al., 2022, emphasis added).

^8^“Part of what [geocentrists] meant by ‘earth' was fixed position. Their earth, at least, could not be moved. Correspondingly, Copernicus' innovation was not simply to move the earth. Rather, it was a whole new way of regarding the problem of physics and astronomy, one that necessarily changed the meaning of both ‘earth' and ‘motion.”' (Kuhn, 1962, pp. 149-50).
